# An Extended UTAUT Model to Explain Factors Affecting Online Learning System Amidst COVID-19 Pandemic: The Case of a Developing Economy

**DOI:** 10.3389/frai.2022.768831

**Published:** 2022-04-28

**Authors:** Gesselle B. Batucan, Gamaliel G. Gonzales, Merly G. Balbuena, Kyla Rose B. Pasaol, Darlyn N. Seno, Roselyn R. Gonzales

**Affiliations:** ^1^College of Education at Danao Campus, Cebu Technological University, Cebu City, Philippines; ^2^Educational Research and Resource Center, Cebu Technological University, Cebu City, Philippines

**Keywords:** COVID-19 pandemic, e-UTAUT, online learning, developing economies, higher education

## Abstract

From a developing country perspective, this study explains the factors affecting online learning amidst the COVID-19 pandemic. The paper empirically tests the proposed extended unified theory of acceptance and use of technology (e-UTAUT) model in the students' intention and use behavior toward the online learning system. Understanding the acceptance of online learning technology is crucial, especially among developing countries caught off-guard by the abrupt transition of face-to-face classes to pure online learning. The enjoyment, interactivity, flexibility, and quality of online learning systems were added as antecedent variables to the UTAUT model. Eight hundred eighty valid responses from selected college students in the Visayas regions, Philippines, were collected. Structural equation modeling (SEM) was employed to verify the research hypotheses. The results supported the proposed model with acceptable fit measures and substantial explanatory power. The extended constructs provide different views on online learning based on the significant cluster of antecedents to explain technology acceptance through behavioral intentions and actual system usage. The paper implies that despite the challenges of connectivity in developing countries, the variations still conform with emerging literature about the topic. Insights for higher education institutions and policy directions are recommended.

## Introduction

The outbreak of COVID-19 changed the landscape of the educational systems and has made the learning institutions shift from the traditional face-to-face to online teaching-learning modality. The changes have set unprecedented transition challenges that are more pronounced among developing economies, highlighting the internet infrastructure as one of the most daring barriers (Costan et al., 2021; Szopiński and Bachnik, 2022). For a developing country like the Philippines, the COVID-19 pandemic has accelerated the adoption of online tools that support remote-based teaching, helping institutions leapfrog into adopting tools that support twenty-first-century learning. Researchers even believe that the pandemic disrupted an education system long lost its relevance and provided an opportunity to introduce digital learning (Pokhrel and Chhetri, 2021). Online learning helps academic managers to go along with the changes by using online platforms that enable teachers and students to continue the teaching-learning process while supporting the students in developing abilities, skills, and attitudes (Vlachopoulos, 2020). Online learning platforms' can cover a course with about 50% less time than face-to-face learning (Li and Lalani, 2020). The online environment supports self-directed learning where students can revisit concepts and set personal goals. For this modality to be effective, there is a need for a comprehensive understanding among learners, instructors, and organizations about its benefits (Adedoyin and Soykan, 2020). As the world responds to the challenges of the pandemic, the learning institutions are also reassessing the acceptance of online learning systems as the global educational environment (UNESCO, 2020).

Emerging literature revealed various aspects of the acceptance of online learning systems. Kim et al. (2021) reviewed the acceptance of online learning using the social psychology theories investigating the mediating impact of user innovativeness amidst the disruption of classes due to COVID-19. The pandemic has created opportunities to reassess the effects of behavioral constructs on the intentions to use and the actual usage of online learning systems. In a survey of 1,009 students from four countries (USA, Peru, Mexico, and Turkey) about the use and acceptance of emergency online learning, cognitive engagement and self-efficacy vary with students' attitudes toward online learning. Understanding the factors affecting online learning amidst the COVID-19 pandemic is essential for the success of technology use. Hypothetically, the acceptance of online learning technology may affect other related latent constructs, especially when the technology has been introduced abruptly due to the COVID-19 pandemic. Recently, university students expressed discontent about the emerging online education amidst the pandemic due to lack of preparations. For instance, 99 percent of the students in Korea, during a survey of 203 Korean universities on a student council network, expressed discontent with online lectures. The main reason for the reported dissatisfaction was the poor quality of online classes, the inability to utilize the school facilities, and difficulty finding a job (Yonhap News, 2020). The structures of this behavioral dismay are best described in a structural equation modeling (SEM) research specifically on the acceptance of technology. However, when online learning became a necessity nowadays, the reality is that online learning is arduous from many developing countries geographically due to the lack of internet and computers services or the inability to afford the high cost of internet access (Qiao et al., 2021). It should be noted how online learning can be improved in the respective of technological evolution if it is the only way for education when facing new influencers, such as COVID-19.

Hassanzadeh et al. (2012) argued that higher education changes with the advent of information technology. The behavioral aspects of the use of technology are important factors. Teo (2011) viewed technology acceptance as a person's willingness to embrace technology to facilitate tasks based on the support it intends to provide. Recently, the acceptance of the online learning system has been examined by researchers in various educational institutions around the world, using multiple models based on distinct criteria. For example, Pham and Dau (2022) revealed that the perceptions of the students in higher education in Vietnam on the uses of online learning system is not an assurance to gain in their performance and that the effort expectancy on online learning readiness has been criticized. These relationships of these factors have been examined using technology acceptance theories such as the technology acceptance model (TAM) and the unified theory of acceptance and use of technology (UTAUT). Other factors such as planning, structural and organizational aspects, the components of a system and the interfaces between them, and various related issues, such as human resources, decision-making, and training, were used to extend the TAM and UTAUT (Anderson, 2008).

The current paper explains students' perspectives on the factors that affect online learning amidst the Covid-19 pandemic by extending the UTAUT model with relevant factors on mandatory e-learning environment (Dečman, 2015). The researchers aimed to empirically test the factors that affect online learning by adding *system enjoyment, system interactivity, system flexibility*, and *system quality* as antecedent variables to the UTAUT model. The theoretical underpinning of this work was based on the work of Venkatesh et al. (2003), and the extended constructs were derived from Nelson et al. (2005), Kulkarni et al. (2006), Barki et al. (2007), Saraf et al. (2007), and Zhang et al. (2008). The extension is based on the idea that the acceptance of online learning is affected by the characteristics of the learning management systems and internet connectivity.

The remainder of this paper is arranged as follows; section research model and hypothesis development provides the hypotheses development based on the proposed model, while section method explains the methodology. Section results and discussion presents the results and discussions of SEM comprising the following steps: (1) the model specification and extraction of factors through confirmatory factor analysis (CFA), (2) the determination of how well the measured indicators represent the specified constructs, and (3) the evaluation of causal model through path analysis. The implications of the findings are offered in section implication, and the paper ends with concluding remarks.

## Research Model and Hypothesis Development

**Figure 1:**
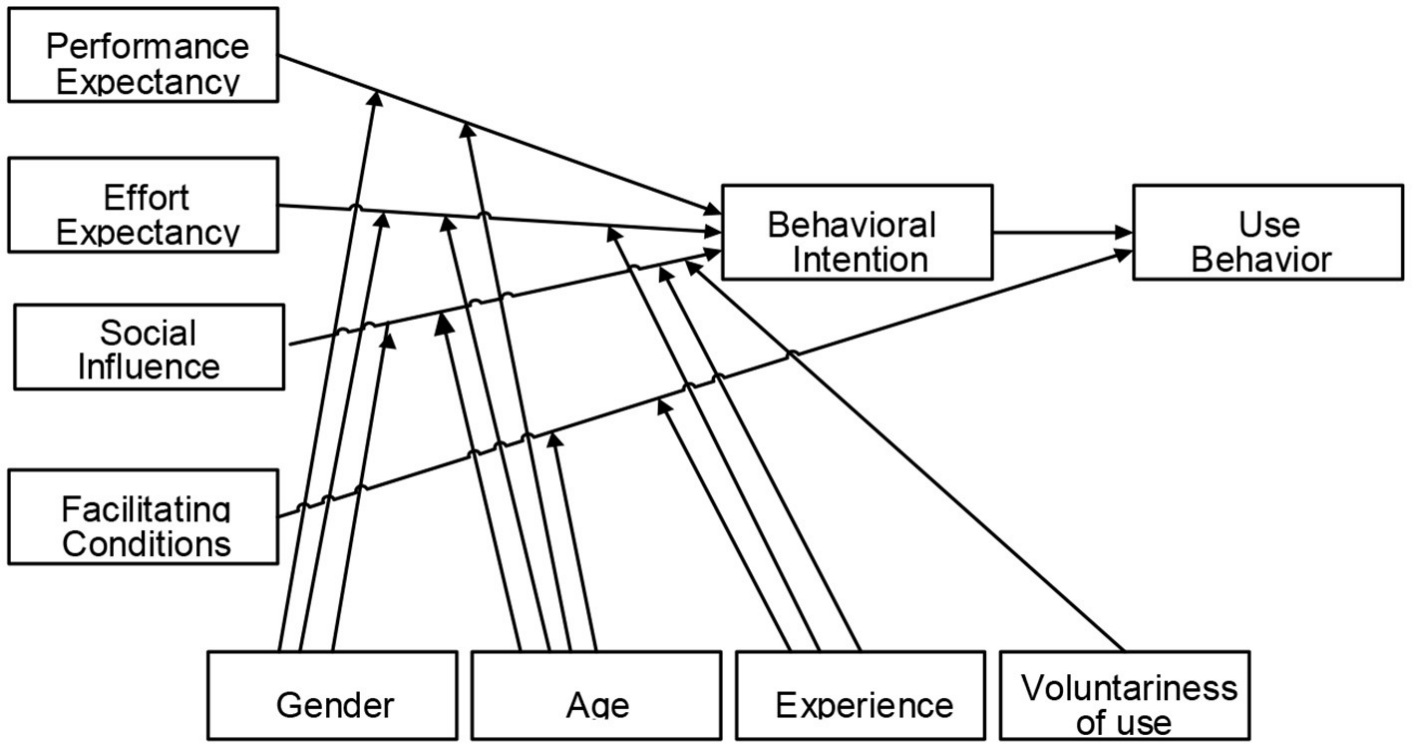
The UTAUT model.

Venkatesh et al. (2003) developed the UTAUT model (see Figure 1), associating the elements of eight models as follows: (i) the theory of reasoned action (TRA) (Ajzen and Fishbein, 1975), (ii) the technology acceptance model (TAM) (Davis, 1989), (iii) the motivational model (MM) (Davis et al., 1992), (iv) the theory of planned behavior (TPB) (Ajzen, 1991), (v) the combined TAM and TPB (C-TAM-TPB) (Taylor and Todd, 1995), (vi) the model of PC utilization (MPCU) (Triandis, 1977; Thompson et al., 1991), (vii) the social cognitive theory (SCT) (Compeau and Higgins, 1995), and (viii) innovation diffusion theory (Rogers, 1995). In this context, the UTAUT model is a theoretical model unifying major theories about information technology acceptance. Many scholars have used various theories/models to examine and predict technology adaptation. The UTAUT model has been used effectively in various studies on technology acceptance and is inferred as a convenient instrument for executives to measure the success of Information technology (Šumak and Šorgo, 2016; Kalavani et al., 2018). Among these theories/models, the authors argue that the new model successfully integrates antecedent variables to better explain the variances in IT *behavioral intention* and *use behavior* in the current situation.

UTAUT is considered a useful and comprehensive model by many researchers as it looks at all of the available theories about technology adoption. Its explanatory power in technology is the highest compared with other technology acceptance theories (Venkatesh et al., 2011). It has also been used to study technological innovations supporting higher education (Halili and Sulaiman, 2018). Specifically, the theory was also used in broad-spectrum educational environments such as virtual learning technologies on a cloud basis, virtual learning environments, desktop web conferencing, and interactive whiteboards (Suki and Suki, 2017). Similarly, education research applied the UTAUT model to highlight the determinants of students' acceptance and use of various technologies in many countries (Khechine et al., 2014). Extended models in UTAUT are also applied to several phenomena, such as the user acceptance of technology in the consumer context (Venkatesh et al., 2012).

The extended unified theory of acceptance and use of technology (e-UTAUT) study develops an integrated model by modifying the UTAUT by adding *use behavior* as an independent variable. Several researchers modified the classical form of the UTAUT model by redesigning the classical model and adding independent variables and determinants or resigning the specific classical determinants and moderators. The exemplary modifications regard new variables or determinants such as *system flexibility* (Hsia and Tseng, 2008), *system enjoyment* (Moon and Kim, 2001), *system interactivity* (Alrawashdeh, 2012), and *system quality* (DeLone and McLean, 1992) many other inclusions of independent variables and moderators. Several UTAUT extensions (variables) and empirical studies were discovered during the literature review. For instance, Nassuora (2013) applied the model by modifying it to add a relationship to understand intent to use online learning. Therefore, this model is a helpful tool for examining students' acceptance of the online learning system during the COVID-19 pandemic.

### System Enjoyment

*System enjoyment* is defined as “the degree of pleasure and enjoyment that users believe they experience while interacting with a given IT system” (Moon and Kim, 2001). Several studies have shown the impact of enjoyment on *behavioral intention* based on specific cases of technology adoption, such as a single platform on mobile payments (Sudono et al., 2020); and online transportation services (Septiani et al., 2017). It was found that enjoyment significantly affects user *behavioral intention* to use an online learning system. Accordingly, Chao (2019) maintained that *system enjoyment* regarding online learning significantly affects *performance expectancy* and *effort expectancy*. Thus, the following are the proposed hypothesis:

H1. *System enjoyment* directly impacts *behavioral intention*.
H2. *System enjoyment* directly impacts *effort expectancy*.
H3. *System enjoyment* directly impacts *performance expectancy*.

### System Interactivity

*System interactivity* refers to the ability to customize the site's look, feel, and content and interact with the user (Palmer, 2002). Through learners themselves and learners' interaction with the organization itself, the interactions between instructors and learners are the critical elements of the learning process (Abbad et al., 2009). The development of technologies used in the online learning context increases individuals' ability to interact from anywhere (Alrawashdeh and Al-Mahadeen, 2013). Abbad et al. (2009) suggested that *system interactivity* indirectly impacts users' intention to use online learning systems through perceived usefulness and ease of use. Consequently, Venkatesh et al. (2003) agree that *performance expectancy* is similar to perceived usefulness, and *effort expectancy* is similar to perceived ease of use. In a study by Compeau and Higgins (1995), the interaction of the high learning system effectively and efficiently increases the perceived usability of a computerized learning system.

Venkatesh (2000) found that the *performance expectancy* also increased as the learning system's experience increased. According to expectancy theory, individual expectations lead to a decision to perform a specific activity. Thus, the student's decision to interact with the learning system depends on their perception of the usefulness of the learning system. Sun et al. (2008) agree that positive interaction of the learning system improves the usefulness of a particular learning system in learners' perception. Thus, the following are the proposed hypothesis:

H4. *System interactivity* directly impacts *effort expectancy*.
H5. *System interactivity* directly impacts *performance expectancy*.

### System Flexibility

*System flexibility* refers to the degree to which a learner believes that they can access the learning system anywhere at any time (Hsia and Tseng, 2008). Arbaugh (2000) suggested that online learning gives students a high degree of flexibility when taking courses online. In other words, learners prefer online learning because of the flexibility of time and place that comes with it. Moreover, flexibility allows students to conveniently manage their learnings, school work, and personal activities (Arbaugh, 2000). The mobile learning environment shows that the perceived flexibility advantages, related to the time and place flexibility, may be closely related to learners' intent to continue learning on mobile devices (Sripalawat et al., 2011). For instance, Evans (2008) suggested that students emphasize flexibility in their *behavioral intention* to adopt mobile learning. Therefore, the following is the proposed hypothesis:

H6. *System flexibility* directly impacts *behavioral intention*.

### System Quality

DeLone and McLean (1992) defined *system quality* as the characteristics that reflect the system's technical level regarding information generation. Tajuddin et al. (2013) showed that *system quality* and users' satisfaction have a positive relationship. Roca et al. (2006) argued that *system quality* improves user satisfaction with technology by encouraging users to use it. Thus, *system quality* is a prominent factor associated with users' satisfaction. Chuan-Chuan Lin and Lu (2000) used the internet to emphasize usefulness on the intention to use online learning. They stated that many people resist using it due to the slow response time despite the internet's popularity. With the website's poor design or merely heavy traffic on the internet, the lack of accessibility of the system induces the availability of related information systems (computers, modems, online services, software, etc.). Therefore, the quality of the information system is considered necessary to influence the user's beliefs of a Web site (Chuan-Chuan Lin and Lu, 2000). Thus, this study strives to test the following hypothesis:

H7. *System quality* directly impacts *behavioral intention*.

### Social Influence

Venkatesh et al. (2003) defined *social influence* as the degree to which individuals perceive that someone accepts that they should use the new system. *Social influence* refers to the students, teachers, friends, classmates, and family members using the online learning system in the educational context. Sripalawat et al. (2011) found that *social influence* is an influential factor in explaining the use of technology. For instance, women are more sensitive to the opinions of others and are therefore more aware of *social influence* when they intend to use new technologies (Venkatesh, 2000). Other literature indicates that *social influence* significantly impacts *behavioral intention* to use online learning (Abu-Al-Aish and Love, 2013). For instance, for young students, the intention to use mobile learning is influenced by the opinion of parents and teachers about the importance of mobile technologies in education. Thus, this study strives to test the following hypothesis:

H8. *Social influence* directly impacts *behavioral intention*.

### Effort Expectancy

The *effort expectancy* construct is the perceived ease of use of the system (Venkatesh et al., 2003). In the online learning context, this variable refers to the students' easiness of using online learning. The relationship between *effort expectancy* and *behavioral intention* was significant and positive. Another study by Alrawashdeh (2012) reported that the relationship between *effort expectancy* and the *behavioral intention* was significant in Jordan's online learning. For instance, the more effort it takes to use technology, the less useful it is perceived to be (Venkatesh, 2000; Venkatesh and Davis, 2000). Accordingly, we propose the following hypothesis:

H9. *Effort expectancy* directly impacts *behavioral intention*.

### Performance Expectancy

*Performance expectancy* is the degree to which student believes that using the system will help them achieve job performance. In the online learning context, this variable refers to the students' study performance. Thus, Venkatesh et al. (2003) showed that *performance expectancy* is the most vital determinant of a user's *behavioral intention* to adopt a technology. Davis (1989) pointed out that *performance expectancy* showed a stronger and more consistent relationship with BI than other variables described in the literature, including various attitude, satisfaction, and perception measures. An additional study by van Dijk et al. (2008) showed that *performance expectancy* and related constructs are the strongest predictors of BI. Another study by Abu-Al-Aish and Love (2013); Chang (2013) suggest that *performance expectancy* positively influences *behavioral intention* to use online learning. For instance, the more individuals expect technology to improve their productivity, the more likely they will use it (Venkatesh, 2000). Accordingly, we propose the following hypothesis:

H10. *Performance expectancy* directly impacts *behavioral intention*.

### Facilitating Conditions

*Facilitating conditions* refers to how an individual perceives that technical and organizational infrastructure is required to use the intended system that is available. Venkatesh et al. (2003), in a study about Users Acceptance of Information Technology, revealed that *facilitating conditions* directly affect *use behavior*. Raza et al. (2021) found that *facilitating conditions* positively affects students' *behavioral intention*. Also, a study by Boontarig et al. (2012) suggested that *facilitating conditions* positively influences the *behavioral intention* and *use behavior* of using smartphones for health services. Based on this discussion, the following hypotheses emerged:

H11. *Facilitating conditions* directly impacts *behavioral intention*.
H12. *Facilitating conditions* directly impacts *use behavior*.

### Behavioral Intention

*Behavioral intention* measures the strength of one's own intention to perform a certain behavior and the willingness of the respondent to use the system (Fishbein and Ajzen, 1977). Warshaw and Davis (1985); Davis (1989) defined *behavioral intention* as a degree to which students formulate a mindful plan to perform specific future behavior and considered as one of the primary dependent variables of the UTAUT model. *Behavioral intention* and *use behavior* are strongly associated, and *behavioral intention* predicts actual *use behavior* (Bhattacherjee and Hikmet, 2008). Also, a study by Venkatesh and Davis (2000) tested that *behavioral intention* assesses the actual *use behavior* of users. Thus, this study strives to test the following hypothesis

H13: *Behavioral intention* directly impacts *use behavior*.

### Gender as a Moderating Variable

Venkatesh et al. (2003) proved that age and gender affect the *behavioral intention* of using technology. The *performance expectancy* was moderated by gender toward *behavioral intention* to use an online learning system. For example, Nysveen and Pedersen (2014) proposed that the effect of *performance expectancy* is stronger for men than for women. Gender differences moderate the effects of *social influence* and the self-management of mobile learning. Age and gender are moderating variables for the relationship between *effort expectancy* and *behavioral intention* (Zhang et al., 2008).

H14a: The impact of *performance expectancy* on *behavioral intention* is moderated by gender.
H14b: The impact of *social influence* on *behavioral intention* is moderated by gender.
H14c: The impact of *effort expectancy* on *behavioral intention* is moderated by gender.

### Use Behavior

Davis (1989) suggested that the *use behavior* construct is often operational by self-reporting participants' degree of current system usage. However, like *behavioral intention, use behavior* was not explicitly defined in the UTAUT model's development, although it measures via system logs (Oh and Yoon, 2014). Thus, Venkatesh et al. (2003) used system logs to provide a logical alternative and may be a preferred method for measuring *use behavior* in research on information systems. Consequently, Venkatesh et al. (2003) suggested that *behavioral intention* significantly influences *use behavior* without assuming a moderation effect between intention and use.

## Method

### Participants

A total of 1,238 College students from the Visayas regions, Philippines participated in the study. In the data quality audit, 358 responses were discarded due to duplication, failure to qualify year level, failure to qualify the scope of the area, and failure to hold sincerity test. Hence, 880 responses were considered valid for further analysis. Table 1 reflects the demographic information of the final participants.

**Table 1:** Demographic information of the participants (*N* = 880).

**Category**		***Total N** **=** **880***	
		***n***	**%**
Gender			
	Male	249	28.3
	Female	631	71.7
Age			
	≤ 20	521	59.2
	21	259	29.4
	21+	100	11.4
Regions in the Philippines			
	6	180	20.4
	7	502	57.1
	8	198	22.5

### Instruments

A questionnaire was created and divided into two parts: (1) the demographic information, (2) the constructs associated with the study.

#### System Enjoyment

The following items measured the *system enjoyment* (Simon et al., 1996; Venkatesh, 2000; Venkatesh et al., 2003). “The online learning systems make school works more attractive.” “I find using the online learning system to be enjoyable.” “The information provided by the online learning system meets my exact needs in learning.” “I find contentment with the accuracy of the online learning system.” “The online learning system provides sufficient information.” The items were measured along a 5-point Likert scale, which ranges from “strongly agree” (1) to “strongly disagree” (5). Cronbach's alpha for the scale was 0.871.

#### System Interactivity

The following items measured the *system interactivity* (Barki et al., 2007): “I use the online learning system (or application) to exchange with other people.” “I use the online learning system (or application) to coordinate” “I use the online learning system (or application) to solve various” “For accomplishing my tasks, an online learning system is essential.” “I use the online learning system (or application) to plan or follow up on my tasks.” The items were measured along a 5-point Likert scale, which ranges from “strongly agree” (1) to “strongly disagree” (5). Cronbach's alpha for the scale was 0.828.

#### System Flexibility

The following items measured the *system flexibility* (Nelson et al., 2005; Saraf et al., 2007): “The online learning system is versatile to meet the needs as they arise.” “The online learning system can flexibly adapt to the new demands and circumstances” “The online learning systems can be adapted to address various learning needs.” “The online learning system is highly adaptable.” “The online learning system is designed to accommodate changes” The items were measured along a 5-point Likert scale, which ranges from “strongly agree” (1) to “strongly disagree” (5). Cronbach's alpha for the scale was 0.862.

#### System Quality

The following items measured the *system quality* (Barki et al., 2001; Kulkarni et al., 2006): “The online learning system allows me to add useful knowledge.” “The online learning system is user-friendly or easy to use.” “The online learning system is accessible from anywhere by anyone.” “The range of functions offered by the online learning system is adequate.” “The information provided by the online learning system is precise.” The items were measured along a 5-point Likert scale, which ranges from “strongly agree” (1) to “strongly disagree” (5). Cronbach's alpha for the scale was 0.791.

#### Social Influence

The following items measured the *social influence* (Venkatesh et al., 2003; Flynn and Ames, 2006): “People who influenced my behavior think that I should use the online learning system.” “I have to use the online learning system because that's how the people who are close to me think.” “People in my organization who use the online learning system have more prestige than those who do not.” “I can direct and guide meetings in my favor in the online learning system.” “I can build effective learning relationships with others in the online learning system.” The items were measured along a 5-point Likert scale, which ranges from “strongly agree” (1) to “strongly disagree” (5). Cronbach's alpha for the scale was 0.726.

#### Effort Expectancy

Derived from Venkatesh et al. (2003); Brown et al. (2010), the *effort expectancy* of the students is measured by the following items: “My interaction with the online learning system is clear and understandable.” “Using the online learning system helps me to become skillful quickly.” “Learning to use the online learning system is easy for me.” “Using an online learning system will not require a lot of mental effort.” “I believe the online learning system (tool) will be easy to use.” The items were measured along a 5-point Likert scale, which ranges from “strongly agree” (1) to “strongly disagree” (5). Cronbach's alpha for the scale was 0.708.

#### Performance Expectancy

The following items measured the *performance expectancy* (Venkatesh et al., 2003, 2012): “The online learning system allows me to achieve the task faster.” “The online learning system increases my work performance.” “I find the online learning system useful for communication.” “I find the online learning system useful in daily life.” “If I use the online learning system, it will increase my chances of getting higher grades.” The items were measured along a 5-point Likert scale, which ranges from “strongly agree” (1) to “strongly disagree” (5). Cronbach's alpha for the scale was 0.778.

#### Facilitating Conditions

Derived from Venkatesh et al. (2003, 2008), we measure students' *facilitating conditions* using the following items: “The guidance from someone helps me in the selection of the online learning system.” “Specialized instructions concerning the online learning system were available to me.” “Using the online learning system fits well with the way I like to deal with my school works.” “I have the resources needed to use the online learning system.” “I am aware of how to use the online learning system. The items were measured along a 5-point Likert scale, which ranges from “strongly agree” (1) to “strongly disagree” (5). Cronbach's alpha for the scale was 0.737.

#### Behavioral Intention

The following items measured the *behavioral intention* (Hong et al., 2002; Malhotra and Galletta, 2005): “I intend to continue using the online learning system in the future.” “I intend to use the online learning system to communicate with others as part of my studies/classes.” “I intend to use the online learning system in doing performance-based activities.” “I intend to use the online learning system for coordinating and collaborating with my classmates.” “I intend to use the online learning system in my daily school activities.” The items were measured along a 5-point Likert scale, which ranges from “strongly agree” (1) to “strongly disagree” (5). Cronbach's alpha for the scale was 0.852.

#### Use Behavior

The following items measured the *use behavior* (Davis et al., 1989; Ajzen, 1991; Venkatesh et al., 2003): “If I had the opportunity to use an online learning system, I would prefer to use it.” “If I can proceed with my schooling using an online learning system, I will.” “I am satisfied with my decision to use the online learning system.” “I use an online learning system to manage my school tasks.” “I will use the online learning system in the future.” The items were measured along a 5-point Likert scale, which ranges from “strongly agree” (1) to “strongly disagree” (5). Cronbach's alpha for the scale was 0.848.

### Data Analysis

**Figure 2:**
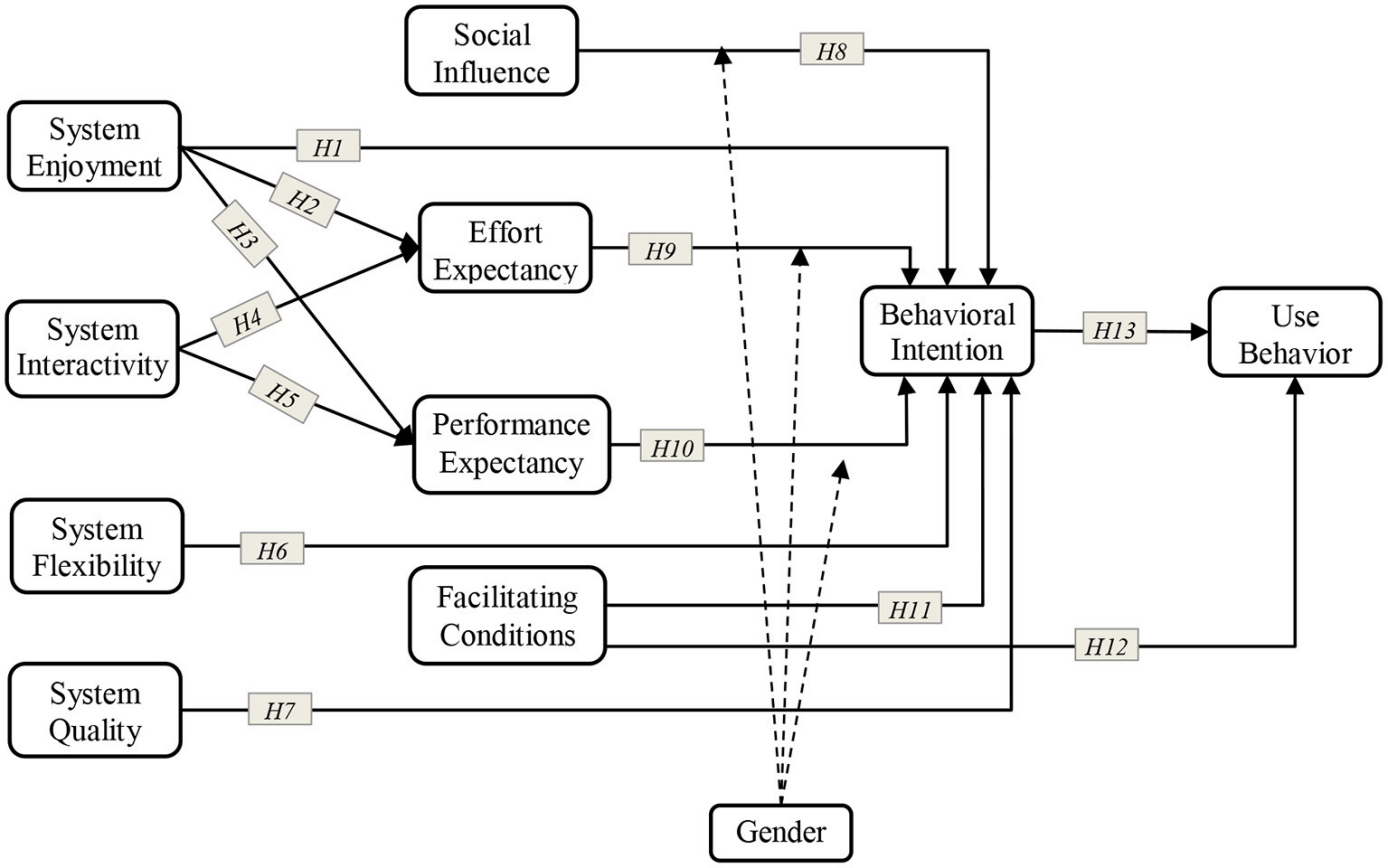
The proposed model.

This study used SPSS software to analyze the items in terms of reliability and validity, while the AMOS 27 software was used to evaluate the measurement model and the path analysis. SEM is a powerful statistical method that simultaneously examines a series of separate multiple regression equations (Pedhazur, 1997). This study evaluated and tested the structural relationship of the UTAUT constructs, as shown in Figure 2.

The reliability of the survey instrument was examined by calculating the Cronbach's alpha of each construct to indicate the internal consistency. Then, convergent validity in this study was examined based on the standard that the estimated coefficient of the indicator was significant. CFA was conducted to assess the measurement model. The three criteria suggested by Fornell and Larcker (1981), that is, standardized loadings, composite reliabilities (CR), and average variance extracted (AVE), were used in this study. These criteria can verify the validity and reliability of the constructs.

The testing of the hypotheses were conducted by path analysis using the SEM approach. We evaluated the structural model of the hypothesized relationships to determine the model's fit. In as much as the overall goodness-of-fit using chi-square is sensitive to large sample size, we alternatively use the minimum discrepancy of chi-square value (CMIN/DF) to evaluate the adequacy of the hypothesized model (Hair, 2009). Other fit indices (i.e., TLI, SRMR, CFI, and RMSEA) were also measured for the sensitivity of the chi-square test to sample size.

## Results and Discussion

**Figure 3:**
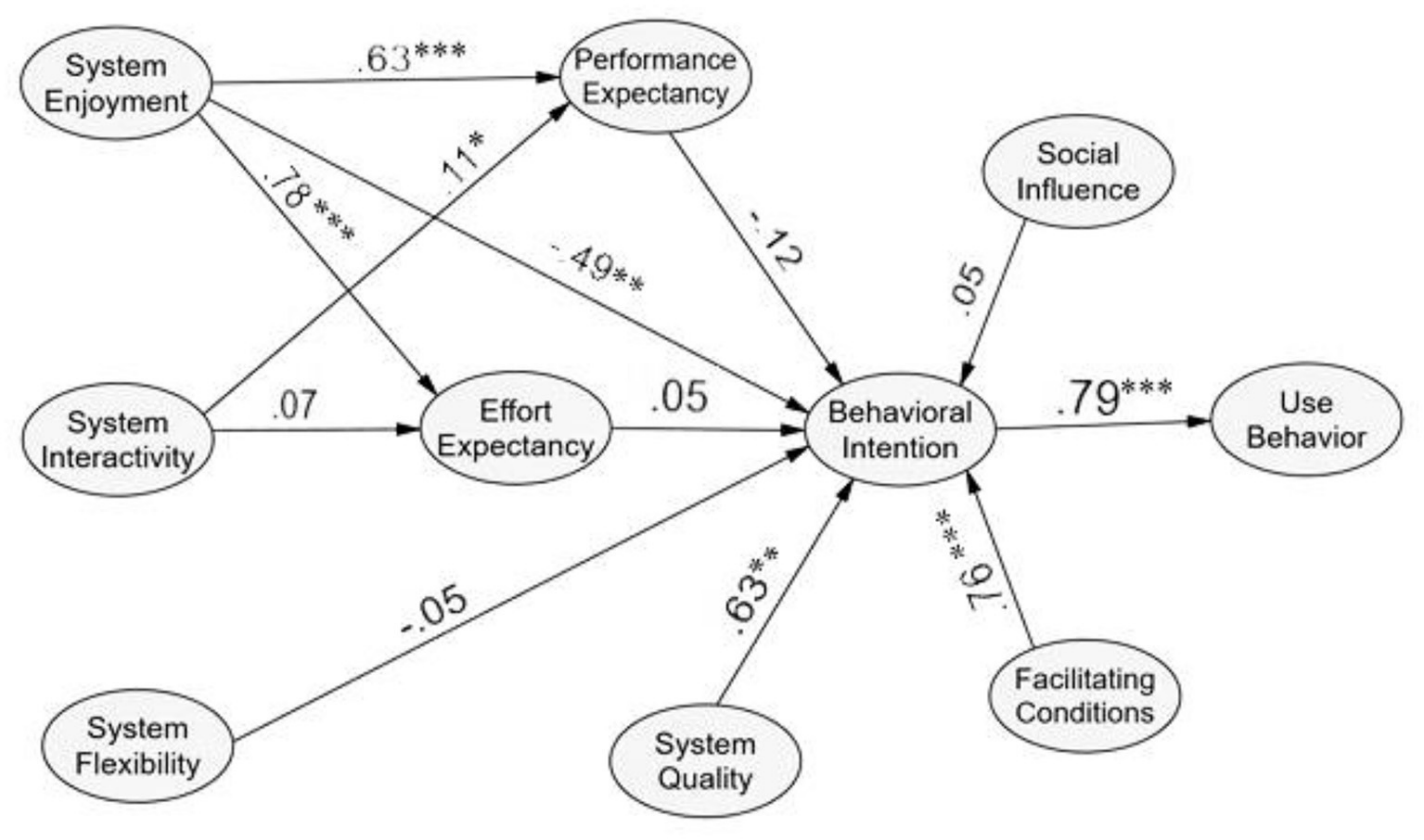
The final study.

This study used a two-step approach to SEM analysis. After conducting CFA to validate the measurement model, the structural model was used to test the hypotheses (see Figure 3).

### Preliminary Analysis

The preliminary analysis is to find the internal reliability indices of each construct using Cronbach's alpha of the original survey items. These indices ranging from 0.708 to 0.871 were reflected in the instrument section. All indices provide a reliable measure of internal consistency (Awang, 2012). Table 2 shows the visual inspection of multicollinearity and discriminant validity using the correlation matrix.

**Table 2:** Zero-order correlations of the study variables.

**Study variable**	**1**	**2**	**3**	**4**	**5**	**6**	**7**	**8**	**9**	**10**
1. BI	1									
2. EE	0.445^**^	1								
3. FC	0.575^**^	0.614^**^	1							
4. PE	0.506^**^	0.637^**^	0.582^**^	1						
5. SE	0.596^**^	0.576^**^	0.561^**^	0.629^**^	1					
6. SF	0.555^**^	0.476^**^	0.544^**^	0.531^**^	0.715^**^	1				
7. SInf	0.542^**^	0.566^**^	0.586^**^	0.576^**^	0.569^**^	0.512^**^	1			
8. SInt	0.559^**^	0.394^**^	0.543^**^	0.462^**^	0.471^**^	0.546^**^	0.456^**^	1		
9. SQ	0.579^**^	0.532^**^	0.576^**^	0.544^**^	0.706^**^	0.709^**^	0.545^**^	0.532^**^	1	
10. UB	0.676^**^	0.498^**^	0.575^**^	0.575^**^	0.681^**^	0.602^**^	0.551^**^	0.554^**^	0.584^**^	1
Mean (x)	2.77	3.02	2.57	2.76	3.17	2.72	2.89	2.45	2.8	3.01
Standard deviation(s)	0.768	0.623	0.585	0.65	0.763	0.685	0.575	0.598	0.67	0.761

*BI, behavioral intention; EE, effort expectancy; FC, facilitating conditions; PE, performance expectancy; SE, system enjoyment; SF, system flexibility; SInf, social influence; SInt, system interactivity; SQ, system quality; UB, use behavior*.

** *p < 0.01*.

The correlation coefficients are all significant at 0.01 (**) alpha levels. The intercorrelations between the constructs ranged from 0.445 to 0.709. The results revealed good discriminant validity since the correlation indices of the study variables are all <0.90 (Lischetzke, 2014). The strongest positive correlation was found between *system quality* and system flexibility (0.709). All other coefficients had moderate correlation ranging from 0.445 to 0.681.

### Measurement Model Results

A total of 880 respondents were loaded to CFA to evaluate the construct validity and measurement reliability. Four measures have been applied to assess the overall metric model fit, namely: the root mean square error approximation (RMSEA), the standardized root mean square residual (SRMR), comparative fit index (CFI), and the Tucker–Lewis index (TLI). Guided by Hu and Bentler (1999), we implement the following cutoff scores to achieve a good model; SRMR must be ≤0.080, RMSEA must be ≤0.060, TLI must be ≥0.900, and CFI must be ≥0.900. Table 3 reflects the standardized loadings, CR, AVE, and Cronbach's alpha of the final model.

**Table 3:** CFA results of the final measurement model.

**Items**	**Standardized loadings**		**CR**	**AVE**	**α**
System enjoyment (SE)	SE1	0.701	0.869	0.571	0.871
	SE2	0.72			
	SE3	0.816			
	SE4	0.798			
	SE5	0.736			
System flexibility (SF)	SF1	0.737	0.863	0.557	0.862
	SF2	0.732			
	SF3	0.773			
	SF4	0.731			
	SF5	0.758			
Behavioral intention (BI)	BI1	0.659	0.856	0.543	0.852
	BI2	0.758			
	BI3	0.752			
	BI4	0.731			
	BI5	0.779			
System interactivity (SInt)	SInt2	0.689	0.808	0.513	0.807
	SInt3	0.663			
	SInt4	0.764			
	SInt5	0.744			
System quality (SQ)	SQ1	0.738	0.755	0.507	0.756
	SQ4	0.712			
	SQ5	0.686			
Use behavior (UB)	UB1	0.725	0.768	0.526	0.787
	UB2	0.688			
	UB5	0.76			
Performance expectancy (PE)	PE1	0.72	0.729	0.574	0.774
	PE2	0.794			
Effort expectancy (EE)	EE1	0.675	0.678	0.514	0.674
	EE2	0.756			
Social influence (SInf)	SInf1	0.825	0.78	0.64	0.808
	SInf2	0.774			
Facilitating condition (FC)	FC2	0.578			
	FC3	0.739	0.608	0.44	0.727

Convergent validity demonstrates in two ways, the factor loadings must be significant and higher than 0.5 (Bagozzi and Yi, 1988), and then, the AVE for each of the factors is >0.5 (Fornell and Larcker, 1981). *Facilitating conditions* has an AVE that is less than the threshold level of 0.5. However, Fornell and Larcker (1981) argued that an AVE of <0.5 is adequate if it bears a CR of higher than 0.6. The reliability of the scale is confirmed because the CR indices of each of the constructs obtained are higher than 0.6 (Bagozzi and Yi, 1988), with levels ranging from 0.608 to 0.869. The overall measurement model showed very satisfactory fit measures of the RMSEA (0.047), SRMR (0.0384), TLI (0.931), and CFI (0.942).

### Relationships Between the Latent Variables

We used the correlational analysis through the Pearson correlation coefficient to support the path analysis of the SEM. The study followed the r-value guidelines (Schober et al., 2018): negligible correlation (0.00–0.09), weak correlation (0.10–0.39), moderate correlation (0.40–0.69), strong correlation (0.70–0.89), and very strong correlation (0.90–1.00).

Table 4 revealed the correlation matrix among the constructs included in the CFA. All correlations were positive and significant at 0.01 alpha level, ranging from 0.30 to 0.729. More specifically, the correlation between *system quality* and *system flexibility* was 0.729, *p* < 0.001, and between *system flexibility* and *system enjoyment*, 0.715, *p* < 0.001, was found to be strong. All other coefficients were found to be moderate, ranging from 0.36 to 0.651. The dependent variable (*use behavior*) was found to be significantly correlated with all nine of the other variables. The correlation of all constructs was higher than the zero-order correlation in the preliminary analysis.

**Table 4:** Correlation results among the constructs in CFA.

**Study variable**	**1**	**2**	**3**	**4**	**5**	**6**	**7**	**8**	**9**	**10**
1. BI	1									
2. EE	0.473^**^	1								
3. FC	0.483^**^	0.458^**^	1							
4. PE	0.390^**^	0.601^**^	0.384^**^	1						
5. SE	0.596^**^	0.598^**^	0.466^**^	0.552^**^	1					
6. SF	0.555^**^	0.494^**^	0.457^**^	0.441^**^	0.715^**^	1				
7. SInf	0.428^**^	0.437^**^	0.400^**^	0.360^**^	0.444^**^	0.397^**^	1			
8. SInt	0.544^**^	0.368^**^	0.457^**^	0.360^**^	0.465^**^	0.538^**^	0.359^**^	1		
9. SQ	0.578^**^	0.531^**^	0.490^**^	0.448^**^	0.718^**^	0.729^**^	0.409^**^	0.556^**^	1	
10. UB	0.651^**^	0.459^**^	0.409^**^	0.445^**^	0.648^**^	0.573^**^	0.448^**^	0.475^**^	0.565^**^	1

*BI, behavioral intention; EE, effort expectancy; FC, facilitating conditions; PE, performance expectancy; SE, system enjoyment; SF, system flexibility; SInf, social influence; SInt, system interactivity; SQ, system quality; UB, use behavior*.

** *p < 0.01*.

### Structural Model

The final model fit measures are acceptable (CMIN = 1372.401, *df* = 459, chi/df = 2.99, CFI = 0.94, TLI = 0.931), whereas RMSEA = 0.048 suggests an excellent fit between the hypothesized model and the observed data (Hair, 2009). The significance of each hypothesized structural path is tested using standardized path coefficients and the *p*-values.

Table 5 showed four paths are significant at *p* < 0.001, two at *p* < 0.01, one at *p* < 0.05 and five paths are not significant. It is noteworthy to mention that H11 (*facilitating conditions* directly impacts *behavioral intentions*) was removed in the final model due to problems on multicollinearity during CFA. The result revealed that *system enjoyment* significantly and inversely affects the *behavioral intention* (β = -0.492, *p* < 0.01), which is a contradiction to the findings of existing literature Alqahtani et al. (2018). This finding adds to the body of literature, specifically in the case of developing economies. In addition, *system enjoyment* directly impacts *effort expectancy* (β= 0.781, *p* < 0.001), and *performance expectancy* (β= 0.634, *p* < 0.001). Chao (2019) demonstrated that perceived enjoyment significantly influenced *performance expectancy* and *effort expectancy* of using mobile learning. Therefore, system enjoyment is a key external variable in the UTAUT model.

**Table 5:** SEM results.

**Hypothesis**	**Path**	**β**	**SE**	**CR**	**Label**
H1	System enjoyment → Behavioral intention	−0.492^**^	0.194	−2.59	Yes
H2	System enjoyment → Effort expectancy	0.781^***^	0.05	13.354	Yes
H3	System enjoyment → Performance expectancy	0.634^***^	0.051	11.439	Yes
H4	System interactivity → Effort expectancy	0.069	0.046	1.453	No
H5	System interactivity → Performance expectancy	0.114^*^	0.051	2.368	Yes
H6	System flexibility → Behavioral intention	−0.047	0.166	−0.295	No
H7	System quality → Behavioral intention	0.631^**^	0.311	2.606	Yes
H8	Social influence → Behavioral intention	−0.047	0.062	0.854	No
H9	Effort expectancy → Behavioral intention	0.045	0.157	0.345	No
H10	Performance expectancy → Behavioral intention	−0.116	0.104	−1.234	No
H12	Facilitating conditions → Behavioral intention	0.764^***^	0.207	3.934	Yes
H13	Use behavior → Behavioral intention	0.79^***^	0.046	21.062	Yes

*** *p < 0.001*,

** *p < 0.01, and*

* *p < 0.05*.

Moreover*, system interactivity* directly impacts *effort expectancy* (β= 0.114, *p* < 0.05). When students intend to use online learning systems to interact with their peers, they also believe that online learning will improve their learning performance. In addition, the result also indicates that the *system quality* directly impacts *behavioral intention* (β = 0.631, *p* < 0.01). Thus, the seventh hypothesis (H7) is confirmed. Therefore, the result shows that *system quality* is considered necessary in affecting the *behavioral intention* of students in online learning.

Furthermore, the twelfth hypothesis (H12) revealed that *facilitating conditions* is positively significant to *behavioral intention* (β = 0.764, *p* < 0.001). This finding has also been confirmed by Sangeeta and Tandon (2021), who indicates that infrastructural support is well-established in schools to facilitate online teaching, and it can enable *behavioral intention*. Moreover, the hypothesis of *behavioral intention* directly impacts *use behavior*. Therefore, H13 is accepted. It means that students who have a higher *behavioral intention* level to use online learning systems will positively influence *use behavior* (β = 0.79, *p* < 0.001). Like Raza et al. (2021), the study concluded a significantly positive link between *behavioral intention* and *use behavior*.

### Analysis of Moderating Effects

The moderating effect of gender on the structural model was analyzed using multigroup analyses. The moderating variable was divided into two groups and analyzed using the critical ratios approach (Byrne, 2010). The comparison of the gender-variable moderator group was split into male (*N* = 249) and female (*N* = 631) respondents.

As shown in Table 6, *social influence, performance expectancy*, and *effort expectancy* were not moderated by gender toward *behavioral intention*. Both males and females do not significantly affect students' intention to use the online learning system. Although not hypothesized, the result showed that gender significantly moderated *system interactivity* to *effort expectancy*. *System enjoyment* is positively significant to *performance expectancy* and *effort expectancy* for both males and females. Also, *use behavior* is moderated by gender toward *behavioral intention*. From our sample, both men and women are college students who do not have the same quality education and access to technology. Therefore, gender did not demonstrate a moderating effect on *performance expectancy, effort expectancy*, and *social influence* on the *behavioral intention* of students' widespread use of technology.

**Table 6:** Effects of moderating variables.

**Gender**	**Male**	**Female**	
	**estimate**	**estimate**	**z-score**
System enjoyment → Effort expectancy	0.632^***^	0.682^***^	0.444
System enjoyment → Performance expectancy	0.478^***^	0.632^***^	1.421
System interactivity → Effort expectancy	0.322^**^	−0.015	−2.81^***^
Social influence → Behavioral intention	−0.001	0.062	0.38
Effort expectancy → Behavioral intention	−0.162	0.161	0.894
Performance expectancy → Behavioral intention	−0.11	−0.131	−0.068
Facilitating conditions → Behavioral intention	0.676	0.904^***^	0.489
Use behavior → Behavioral intention	1.088^***^	0.923^***^	−1.412

*** *p < 0.001 and*

** *p < 0.01*.

## Implication

The results of this study showed several implications. First, the e-UTAUT model makes it relevant to the present situation caused by the Covid-19 and its application in higher education to explain factors affecting online learning, most especially from the experiences of a developing economy. This inference is based on the significance of system enjoyment to intentions to use, the expected effort, and the expected performance of the online learning system. These findings supported Audet et al. (2021), which states that students' adjustment to online learning amidst the COVID-19 pandemic is engaging. Hence, the advantage of using an online learning system in pandemics where institutions are closed are supported with reasonable factor loadings implying flexibility of the students to respond to the situational crisis. The results suggest that higher education institutions build a stable online portal where teachers can teach and guide students without any difficulties.

Secondly, the perceived interactivity and quality of the online learning system significantly explains the students' belief to perform better and, consequently, add to their willingness to use the system. This supports the findings that higher education students are still abreast of digitizing their activities despite being challenged by technological infrastructure in developing economies and actively aspire to develop their technological knowledge (Gonzales and Gonzales, 2021). The clear advantage of system interactivity and quality is that it allows a consolidated variety of information combined. It permits us to store all information in one place, and students can locate them anytime, using compatible devices. It reduces administrative hassles related to maintaining learning materials in multiple areas.

Lastly, the behavioral aspects that facilitate the desire to use the online learning system significantly explain the students' intentions. Thus, this is a reason to believe that this are facilitated with the availability of specialized instructions, awareness, and enough guidelines concerning online learning systems (Yates et al., 2021). The use of an online learning system through successful implementation is recommended to help students examine the benefits of technology. Thus, the utilization of the system is proof that it can make other educational learning activities done online.

## Conclusion

Amidst the COVID-19 pandemic, educational institutions use online learning to meet the needs of students. The complexity of the learning environment in online learning constrains the need to investigate critical latent factors in understanding the usage behavior. The paper extended the UTAUT with enjoyment, interactivity, flexibility, and quality. It is believed that these factors differ among developing economies.

The results revealed that the model had high internal consistency and reliability, indicating that the proposed model possesses substantial explanatory power. This study shows that intention is a key factor that significantly influences students' *use behavior* toward online learning. Students' *system enjoyment* played an important factor in affecting *performance expectancy* and *effort expectancy*. The significance of the negative effect of *system enjoyment* to *behavioral intention* suggests that there is a need for further investigation on the contrariety of the results in developing economies. The significant effect of *system quality* in *behavioral intention* indicates that despite the challenges of connectivity in developing countries, the variations still conform with emerging literature about the topic. Finally, the positive effect of *facilitating conditions* on *behavioral intention* could be attributed to the technical and organizational infrastructure. For example, specialized instructions on online learning and the resources needed were available. Determining what motivates online learning can enrich learning quality and facilitate pedagogical and instructional uses of online learning. Therefore, this study will have significance for decision-makers in higher education institutions.

In the future, it is recommended that the model should be extended to encompass additional constructs, such as system satisfaction and confirmation, along with various moderating variables (i.e., age, experiences, and voluntariness of use). The study also recommends exploring further the variables or indicators of online learning acceptance on usage behavior concentrated on the digital education revolution. The model will then be integrated into an application that will support the growth of technology in education. This can provide a step forward to digital education and technology-rich learning.

## Data Availability Statement

The original contributions presented in the study are included in the article/supplementary material, further inquiries can be directed to the corresponding authors.

## Ethics Statement

The studies involving human participants were reviewed and approved by Local Research Ethics Committee, Cebu Technological University-Danao Campus. The patients/participants provided their written informed consent to participate in this study.

## Author Contributions

GB: project leader, revising the research paper, and analyzing data. GG: research adviser. MB: writes the methods and implications. KP: writes the review of related literature. DS: technical writer and writes the introduction. RG: revising research paper. All authors contributed to the article and approved the submitted version.

## Funding

This work was funded under the Student Trust Fund (STF) the Cebu Technological University Danao Campus, Danao City Cebu, Philippines.

## Conflict of Interest

The authors declare that the research was conducted in the absence of any commercial or financial relationships that could be construed as a potential conflict of interest.

## Publisher's Note

All claims expressed in this article are solely those of the authors and do not necessarily represent those of their affiliated organizations, or those of the publisher, the editors and the reviewers. Any product that may be evaluated in this article, or claim that may be made by its manufacturer, is not guaranteed or endorsed by the publisher.
